# Biodegradation of feather waste keratin by a keratinolytic soil fungus of the genus *Chrysosporium* and statistical optimization of feather mass loss

**DOI:** 10.1007/s11274-016-2177-2

**Published:** 2016-11-24

**Authors:** Justyna Bohacz

**Affiliations:** Department of Environmental Microbiology, Laboratory of Mycology, Faculty of Agrobioengineering, University of Life Sciences in Lublin, 7 Leszczyńskiego Street, 20-069 Lublin, Poland

**Keywords:** Biodegradation, *Chrysosporium* sp., Keratinolytic activity, Keratin waste, Mineral products of keratinolysis

## Abstract

This paper assesses the ability of strains of *Aphanoascus fulvescens* and *Chrysosporium articulatum* isolated from soil (phaesol) to degrade native feather keratin. Strains were identified based on phenotypic traits and nucleotide sequencing. Response Surface Methodology was used to optimize cultivation conditions exhibiting the highest keratinolytic activity. The experiments were based on Box-Behnken designs for the loss of substrate mass (chicken feathers). While substrate mass loss is an “economic coefficient” that reliably indicates feather keratin degradation, it has not been studied before. Stationary liquid cultures of five selected strains were conducted in laboratory conditions at 28 °C using poultry feathers (1 g) as the sole source of carbon, nitrogen and energy. Enzymatic activities, keratin mineralization products and substrate mass loss were determined periodically. The mineralization of keratin proteins by strains yielded a high number of ammonium ions alkalinizing the medium. Increased ammonium ions inhibited the activity of caseinian protease and keratinase. A decrease in the concentration of these ions induced proteolytic enzymes, chiefly the activity of keratinase, at the end of fungal cultivation. Keratinase activity was related to protein- and peptide release and that of caseinian protease to sulfate ions. The highest loss of substrate mass in comparison to the reference strain CBS104.62 (35.4%) was recorded for *Aphanoascus fulvescens* B21/4-5 (65.9%). Based on a Box-Behnken design, the maximum loss of substrate mass for the *Aphanoascus fulvescens strain* (71.08%) can be achieved at pH 7.58 and temperature 28.7 °C.

## Introduction

Keratin waste is high in protein rich in nitrogen and sulfur. It contains 90% of protein, chiefly keratin, and from ~15–18% of nitrogen and 2–5% of sulfur (Kunert 2000; Korniłłowicz-Kowalska 1997a; Onifade et al. 1998). Considerable amounts of this waste, such as feathers, bristles, horns and hooves and similar cornified skin products, are generated in animal raising and trading Korniłłowicz-Kowalska and Bohacz (2011). Keratin proteins have high mechanical resistance and are resistant to chemical agents and enzymatic lysis (proteases). The resistance is attributed to the presence of numerous disulfide bonds (S–S) (Korniłłowicz-Kowalska 1997b). As well as a few insects (moths), only keratinolytic microorganisms have the ability to degrade native keratin in nature. Those include some proper bacteria of the genera *Bacillus, Vibrio, Serratia* (Kim et al. 2001; Grazziotin et al. 2006; Khardenavis et al. 2009), actinomycetes (Vasileva-Tonkova et al. 2009), recombinant strains such as the genus *Bacillus* (Haddar et al. 2009), microfungi specialized in the degradation of that protein (Farag and Hassan 2004; Raju et al. 2007), representing so-call geophilic dermatophytes and related to them fungi known as chrysosporia (deriving the name from the genus *Chrysosporium*). Some species are parasites of keratinized human and animal tissues and cause dermatophytoses. Those include anthropophilic dermatophytes such as *Trichophyton rubrum* and zoophilic dermatophytes such as *Trichophyton verrucosum*, which cause animal infections possibly transferrable to humans. Other keratinophilic fungi, that is geophilic dermatophytes, and so-called chrysosporia are saprotrophs living in keratin-rich dead animal remains depositing in the soil (Korniłłowicz-Kowalska and Bohacz 2011). Three groups of species of geophilic dermatophytes are distinguished. These are: saprotrophic species represented primarily by *Trichophyton terrestre* Durie et. Fray and *T. georgiae* Varsavsky et. Ajello, often pathogenic species, i.e. *Microsporum gypseum* (Bodin) Guiard et. Grigoriakis and *M. fulvum* Uriburu, randomly pathogenic species, i.e. *Microsporum cookei* Ajello and *Trichophyton ajelloi* (Vanbreuseghem) Ajello (Simpanya 2000; Korniłłowicz-Kowalska and Bohacz 2011).

Soil enrichment in keratin is the chief factor determining the occurrence and development of these microorganisms. As well as the nutrient factor, the development of keratinophilic fungi in soil is supported by a high humus content, neutral or slightly alkaline pH reaction, richness in CaCO_3_ (Garg et al. 1985; Korniłłowicz-Kowalska and Bohacz 2002). The *Chrysosporium* group belongs to this group of microorganisms, specialized in the decomposition of native keratin, i.e. feathers, hairs (Kushwaha 2000), whose biodegradation mechanism has received little attention. Although considered safe during certain development stages, these fungi can convert into pathogenic forms under defined environmental conditions. Kushwaha (2000) reports that microfungi of the genus *Chrysosporium* are known to be effective keratin destruents; however, keratin degradation varies depending on the species. The author reports that *A. fulvescens* and *A. verrucosus* were found to penetrate the hair structure differently from *A. keratinophilus*.

The aim of this study was to assess the dynamics of the biodegradation of feather keratin by five strains of the genus *Chrysosporium* isolated from the soil based on their keratinolytic activity, loss of feather mass and keratin mineralization products, i.e. N–NH_4_ and S–SO_4_. Fungi were identified using phenotypic traits, PCR analysis and nucelotide sequencing. Response Surface Methodology (RSM) was used to optimize cultivation conditions for enhancing the biodegradation process of feather waste by the strain exhibiting the highest keratinolytic activity. The experiments were based on a Box-Behnken design for the loss of substrate mass (chicken feathers). Substrate mass loss is an economic coefficient and a reliable indicator of feather keratin degradation but has not been optimized. Such optimization has been performed for keratinase activity in many studies (Anbu et al. 2005, 2007; Liang et al. 2011).

## Materials and methods

### Soil

Five keratinophilic fungi (1-B28/6, 2-B21/4-5, 3-B25/1, 4-B42/4, 5-B26/2) examined in this study were isolated from pheosols (acc. to FAO). The arable layer (0–20 cm) of soil in an arable field (Bezek village, 57.4 km from Lublin, 51^o^12′N 23^o^16′E, Lublin region, central-eastern Poland) was sampled. Soil properties are discussed in a study by Bohacz and Korniłłowicz-Kowalska (2012).

### Isolation of *Chrysosporium* fungi

Keratinophilic fungi were isolated using the keratin bating method (Simpanya and Baxter 1996) with white chicken (broiler chicken) feathers as the substrate. Chicken feathers were sourced from a poultry company (Indykpol, Lublin, Poland). Feathers were washed, thoroughly rinsed with distilled water, dried, broken up by manual cutting into 0.5 cm fragments and autoclaved [121 °C, 100 kPa (15psi), 30 min.]. Plates containing soil material and sterile feathers were incubated in a humidity chamber at 26 °C. Growing mycelia were plated onto Sabouraud glucose agar containing actidion and chloramphenicol after 4–6 weeks.

### Morphological tests of fungal strains

Pure cultures were identified to the genus and species level based on macroscopic observations on plates and microscopic observations in microcultures (an Olympus BX-41 laboratory microscope fitted with a CVIII4 digital camera integrated with a computer equipped with Cell-A software for image analysis, reporting and archiving) using specialist systematic studies by Domsch et al. (1980) and van Oorschot (1980). Species identification was conducted on the basis of morphological (phenotypic) traits. It was performed in microcultures on agar discs (microscopic observations) and the following traits were examined: racquet hyphae present or absent, aleurioconidia, position (terminal and/or lateral sessile on short protrusions, solitary or in short chains); arthroconidia: shape (cylindrical or barrel-shaped). The following characters were examined in macroscopic observations of cultures on plates and slants on Sabouraud medium (without antibiotics): colony size (after 7 and 14 days); averse and reverse pigmentation, colony structure (felty or powdery) and margin (regular or fimbriate). Final species identification was compared to the reference strains obtained from the Centraalbureau voor Schimmelcultures (CBS): *Chrysosporium keratinophilum* (CBS 104.62.99.5196) and *Ch. queenslandicum* (CBS 280.77).

### DNA extraction, PCR amplification and sequencing

Fungal species were identified using ITS (internal transcribed spacer) region sequencing method. The total genomic DNA was extracted from mycelium of the fungi using CHELEX resin (Biorad) and enzymes for digesting the cell wall, i.e. lyticase (1 mg/1 ml) and Proteinase K (20 mg/ml). The entire ITS region was amplified using two primers: ITS1 (5′ TCCGTAGGTGAACCTGCGG′3) and ITS4 (5′TCCTCCGCTTATTGATATGC′3) (White et al. 1990). Reactions were done in 20 µl volumes with final concentrations of reactants as follows: 2 µl buffer 10 × B dedicated for PCR from the OptiTaqEurx set with MgCl_2,_ 0.4 µl 10 mM dNTPs, and 0.4 µl 10 µM of each primer ITS1 and ITS4, 0.2 µl OptiTaq polymerase (Eurx, Gdańsk, Poland) (1U per reaction), 14.4 µl miliQ water and 2 µl of DNA template. Cycling parameters were 95 °C for 2 min followed by 35 cycles of 95 °C for 15 s, 55 °C for 15 s and 72 °C for 35 s, and the final extension at 72 °C for 7 min. Cycle sequencing reactions were performed using purified PCR products using BigDye Terminator Mix v. 3 (Applied Biosystem) according to the manufacturer’s instructions. The ITS was sequenced bidirectionally using PCR primers. The products were resolved by capillary electrophoresis using the Applied Biosystems Inc. ABI3730xl DNA genetic analyser in the DNA Sequencing and Oligonucleotide Synthesis Laboratory, Institute of Biochemistry and Biophysics oligo.pl, Polish Academy of Sciences, Warsaw, Poland.

### Sequence analysis

Contiguous sequences (contigs) were assembled from chromatogram sequence reads using Seqman (DNAStar) and a consensus sequence was generated. A BLAST search of the sequences was performed against NCBI-GenBank database for comparison.

The nucleotide sequences are available in GenBank database under accession numbers i.e. KY014757 (strain B21/4-5), KY014758 (strain B25/1), KY014759 (strain B26/2), KY014760 (strain B28/6), KY014761 (strain B42/4).

### Fungal cultures

The experiment was carried out in stationary liquid medium containing native chicken feathers as the sole source of C, N, S and energy at 28 °C. Feathers were sterilized with the gas method and combined with a sterile mineral medium containing K_2_HPO_4_-1.5; NaCl-0.01; MgCl_2_^·^7H_2_O-0.05; H_2_O-1000 cm^3^; pH-6.5 as described in a study by Korniłłowicz (1994). Cultures were conducted in 300 cm^3^ Erlenmeyer flasks containing 100 cm^3^ of mineral substrate with 1 g of feathers inoculated with 1 cm^3^ of spore suspension at a density of 10^7^–10^9^ cfu cm^−3^. A mineral-keratin medium that had not been inoculated with fungal spores was the control. The experiment was carried out in three replicates.

### Chemical and biochemical analyses in post-culture filtrates

Periodical biochemical and chemical analyses were conducted. The concentration of soluble protein and released peptides (pr) was determined using the Lowry method modified by Schacterle and Pollack (1973). The content of ammonium ions (NH_4_) was established by nesslerization using 410 nm wave lengths as described by Korniłłowicz-Kowalska (1997b) and the content of sulfate ions (SO_4_) by nephelometry using wave lengths at 490 nm as given in a study by Korniłłowicz-Kowalska (1997b). Keratinase activity (KA) was determined according to the method of Yu et al. (1968) as modified by Anbu et al. (2005). Protease activity (PA) was measured according to Korniłłowicz (1994). Substrate use was estimated by determining loss of its dry mass by weight at 105 °C. The pH of post-culture liquids was determined using a pH-meter with a glass electrode (pH-meter CP-501, Elmetron).

A reference strain, *Chrysosporium keratinophilum* CBS 104.62, was used to demonstrate the efficiency of chicken feather degradation (loss of substrate mass) by the strains. *Ch. keratinophilum* is defined as an alternate state of *Aphanoascus fulvescens* (teleomorph) in the ATCC database.

The latter is consistent with the data given by van Oorschot, who revised the genus *Chrysosporium* (van Oorschot 1980). Van Oorschot reports that *Chrysosporium keratinophilum* is an anamorph of *Aphanoascus fulvescens* (teleomorph).

### Statistical results analysis

Statistical analyses were conducted with STATISTICA 12. The Fisher test at the significance level α = 0.05 and statistically homogenous groups were used to assess the significance of the difference between keratinolytic activities of the fungal strains and also between the study periods. The Principal Component Analysis is used to reduce the number of variables and to explain the correlations between them (protease activity, keratinase activity, level of released proteins, ammonium ions and sulfate ions, pH of the medium in this study) with two unobserved and uncorrelated principal components. The regression functions (polynomial of degree two) were determined for variability of the traits under examination in potentially keratinolytic fungi.

### Experimental design

In order to improve cultivation conditions of keratinolytic fungi of the genus *Chrysosporium* against biodegradation of feathers, Response Surface Methodology (RSM) was used. Strain B21/4-5 which showed high keratinase activity and high loss of substrate mass was selected. As reported by Korniłłowicz-Kowalska (1997a), the loss of substrate mass is the most reliable indicator of keratinolytic activity of strains of keratinolytic fungi. Optimization conditions were designed based on two strains belonging to the genus *Chrysosporium* (Kushwaha 2000). The experimental design and statistical analysis were performed using Statistica 12. The experiments were based on a Box-Behnken design (Box and Behnken 1960) with a quadratic model in order to study the interactions of three independent variables: medium pH, temperature (^o^C), and the amount of feathers (g). The tree selected variables were represented by *X*_1_, *X*_2_, *X*_3_, respectively. Each independent variable was coded in three factor levels which were −1 (low), 0 (central point) and +1(high) as shown in Table 1.

**Table 1:** Independent variables and their coded levels used for the optimization of loss mass substrate by the keratinolytic fungus

Factors	Independent variables	Range levels		
		Low	Medium	High
		−1	0	+1
X_1_	pH	6.5	7.5	8.5
X_2_	Temperature (°C)	25	28	37
X_3_	Feathers (g)	0.5	1	2

Optimization experiments were based on 15 combinations with three replicates. As shown in a study by Anbu et al. (2007), the relationship was calculated by the second order polynomial Eq. (1)

1 $$Y = R_{0} + \sum {R_{i} X_{i} } + \sum {R_{ii} X_{i}^{2} } + \sum {R_{ij} X_{i} X_{j} + \varepsilon }$$

where *X*_1_ *, X*_2_ *, X*_3_… are the input variables which affect the response *Y, R*_0_ *, R*_*i*_ *, R*_*ii*_ and *R*_*ij*_ are the know parameters, ɛ is the random error.The number of experiments required for the development of a Box-Behnken design were defined as (2)

2 $$N = 2k + (k - 1) + C_{0}$$

where *N*-number of experiments, *k*-number of factors, *C*_*o*_-number of central points.

A statistical analysis of the model was performed to evaluate the analysis of variance (ANOVA). The quality of the polynomial model equation was judged statistically by the coefficient of determination R^2^, and its statistical significance was determined by an F-test (Statistica 12 software, Poland).

## Results

### Sequence characteristics

The consensus sequence of the ITS fragment (query) was submitted for comparison with the sequence deposited in GenBank (subject). A 100% coverage was found for all the species. 100% similarity to *Chrysosporium articulatum* (strain B25/1) was detected, 99% similarity to *Aphanoascus fulvescens* and *Chrysosporium articulatum* (strains B21/4-5, B28/6, B42/4) as well as *Aphanoascus terreus* and *A. fulvescens* (strain B26/2) (Table 2).

**Table 2:** Results of molecular identification of fungi

	Fungal species		Similarity (%)	The highest sequence similarity (%)
1	B28/6	*Aphanoascus fulvescens* NBRC31723 or *Chrysosporium articulatum* strain UOA/HCPF9038 isolate ISHAM-ITS_ID MITS1 141	99	100
2	B21/4-5	*Aphanoascus fulvescens* NBRC31723 or *Chrysosporium articulatum* strain UOA/HCPF9038isolateISHAM-ITS_ID MITS1141	99	100
3	B25/1	*Chrysosporium articulatum* UAMH4320	100	100
4	B42/4	*Aphanoascus fulvescens* NBRC31723 or *Chrysosporium articulatum* strain UOA/HCPF9038 isolate ISHAM-ITS_ID MITS1 141	99	100
5	B26/2	*Aphanoascus fulvescens* NBRC31723or *Aphanoascus terreus* isolate 29MIBA191NPKJ59759AT	99	100

### Morphological traits of fungal strains

Morphologically, strains of *A.fulvescens* have the following traits: reaching 32–37 mm in diameter after 7 days on Sabouraud medium at 28 °C and 55–80 mm diameter after 14 days, racquet hyphae present, terminal and lateral conidia, smooth-walled conidia, one-celled, intercalary conidia less abundant, reverse pale creamy yellow, averse white and sulfur yellow (all strains).

The morphology of the strains seems to be very similar to that of the anamorphic state of *Chrysosporium keratinophilum*. In accordance with the latest nomenclature (Index Fungorum) currently in use (http://www.indexfungorum.org/), the species name of the fungus is the name of the perfect stage (teleomorph), i.e*. Aphanoascus keratinophilus*.

*Chrysosporium keratinophilum* (anamorph) is listed as an alternate state of *Aphanoascus fulvescens* (Cooke) Apinis (teleomorph) in the ATCC American collection of fungal strains.

The strain belonging to *Ch. articulatum* was characterized by the abundance of cylindrically shaped intercalary conidia under the microscope. Terminal and lateral aleurioconidia were less numerous, sessile or on short protrusions, usually single, smooth, clavate. Colonies reaching 32–37 mm in diameter after 7 days on Sabouraud medium at 28 °C and 70–90 mm in diameter after 14 day. Reverse pale creamy yellow and averse white, slightly fluffy.

Based on the analysis of the genetic material, the macro- and microscopic traits and the analysis of the ATCC and CBS culture collections, four keratinolytic fungi (1-B28/6, 2-B21/4-5, 4-B42/4 and 5-B26/2) were identified as strains of *Aphanoascus fulvescens* (teleomorph) („alternate state *Chrysopsorium keratinophilum*—anamorph; cited after ATCC). The strain designated as 3-B25/1 was identified as *Chrysosporium articulatum* (MycoBank synonym *Chrysosporium queenslandicum*). All strains are available in Department of Environmental Microbiology, Faculty of Agrobioegineering, University of Life Sciences in Lublin, Poland.

### Substrate utilization

**Fig. 1:**
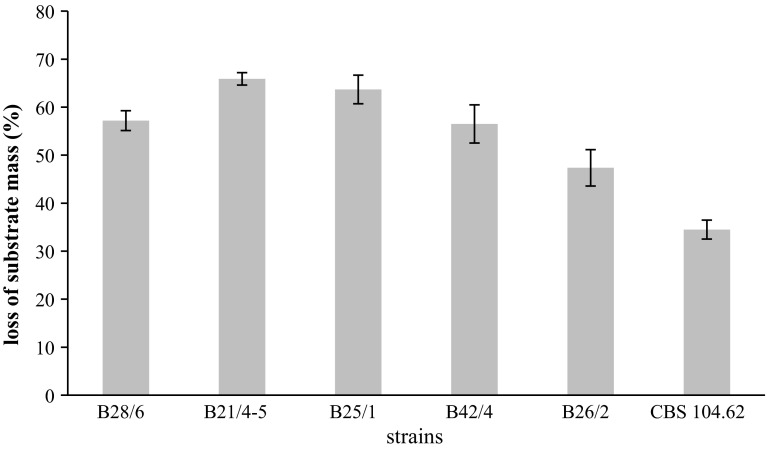
Loss of substrate mass (%) on day 42 by *Chrysosporium* strains. *Error bars* indicate standard deviation (n = 3)

Abilities of saprotrophic fungi to degrade native keratin were assessed based on the utilization of native chicken feathers. All the strains (five isolates) utilized feathers as the sole source of C, N, S and energy. Of these fungi, *Chrysosporium articulatum* and *Aphanoascus fulvescens* strains designated as 3 and 2, (B25/1 and B21/4-5, respectively), were the most active destruents of feather keratin (63.7 and 65.9% substrate mass loss after 42 days of cultivation, respectively). The reference strain *Chrysosporium keratinophilum* CBS 104.62 exhibited a weaker keratinolytic activity and the percentage of feather mass loss was lower by half (35%) (Fig. 1).

### Release of soluble proteins and peptides

Soluble proteins and peptides were released in the cultures of all the strains. Their levels increased together with culture time peaking on day 42 (Table 3). As evidenced by the values of the coefficient of determination (R^2^ = 54.54%), protein release varied among the fungal strains during keratin degradation. A statistical analysis of homogeneous groups showed that the strain designated as 1 (B28/6) differed significantly from other strains, especially from 3 (B25/1) and 2 (B21/4–5) strains which were the best degraders (Table 3).

**Table 3:** Changes in the release of soluble proteins and peptides (µg. cm^−3^ of the post-culture medium) in the cultures of *Chrysosporium* strains; values means of three replicates ± standard deviation

Times of analyses (days)	Strains of fungi					Means for time
	1	2	3	4	5	
7	120.53aB	148.69abE	162.34bD	130.45abC	127.89abB	137.98d
	±*24.84*	±*3.03*	±*21.27*	±*6.30*	±*14.00*	
14	142.08aB	191.52bA	192.42bE	161.49aD	158.93aB	169.23e
	±*21.96*	±*2.79*	±*3.45*	±*1.84*	±*12.10*	
21	48.42bD	88.96aD	88.74aC	64.21abB	69.22abC	71.91c
	±*15.82*	±*19.53*	±*20.29*	±*2.64*	±*7.07*	
28	178.24bAC	205.97abAB	209.49abA	224.53aA	232.53aA	210.15a
	±*3.85*	±*1.06*	±*3.57*	±*31.42*	±*19.77*	
35	190.50bA	224.74aBC	226.88aAB	226.88aA	241.92aA	222.18ab
	±*14.35*	±*5.36*	±*1.81*	±*7.95*	±*22.28*	
42	204.81bA	237.65abC	245.12aB	245.54aA	254.72aA	237.57b
	±*11.76*	±*7.06*	±*3.77*	±*8.00*	±*31.24*	
Means for experimental treatment	**147.43A**	182.88AB	194.11B	176.85B	172.92AB	

1- B28/6, 2- B21/4-5, 3- B25/1, 4- B42/4, 5- B26/2

Small letters (a, b, c, d, e) are used to designate homogenous groups in a specific period for all the strains (analyzed in rows). Capital letters (A, B, C, D, E) are used to designate homogenous groups for individual strains in the experimental periods analyzed (analyzed in columns)

The same letters (among a, b, c, d, e, A, B, C, D, E) were used to designate those means that form homogenous groups. This means that if two means are given the same letter (e.g. a, A), then the means do not differ from each other significantly (significance level α = 0.05). However, if a pair of means is given different letters (e.g. a and b; A and B), then the means differ significantly from each other (for the same significance level α = 0.05)

### Proteolytic and keratinolytic activities of fungi degrading feather waste

Extracellular proteases active against casein were detected in raw post-culture filtrates of all the strains examined. Enzyme secretion was high up to day 14 of cultivation. After that, the activity decreased up to day 28 and again increased up to day 42 of cultivation. However, the level of the coefficient of determination was low (R^2^ = 36.4%) and proteolytic activity varied considerably among the fungal strains, with strains 4 and 5 differing significantly (Table 4).

**Table 4:** Changes in the activities of extracellular keratinase (KA) (U. cm^−3^ of the post-culture liquid) and protease (PA) (µg of tyrosine. cm^−3^ of the post-culture liquid) in the cultures of *Chrysosporium* strains; values means of three replicates ± standard deviation

Times of analyses (days)	Strains of fungi					Means for time
	1	2	3	4	5	
*Keratinase activity* (*KA*)						
7	1.29aA	0.98aA	0.95aA	1.58aA	1.5aAB	1.26a
	±*0.09*	±*0.19*	±*0.36*	±*0.68*	±*0.29*	
14	1.96aB	2.15aA	2.15aC	1.70aA	2.59aC	2.11b
	±*0.68*	±*0.24*	±*0.42*	±*0.15*	±*0.49*	
21	1.08aA	1.98bA	1.25aAB	1.62abA	1.58abAB	1.50ab
	±*0.15*	±*0.20*	±*0.43*	±*0.39*	±*0.5*	
28	1.06aA	1.25aA	1.21aAB	1.10aA	0.97aA	1.11a
	±*0.01*	±*0.48*	±*0.20*	±*0.25*	±*0.22*	
35	1.62aAB	1.70aA	1.78aBC	1.51aA	2.07aBC	1.73ab
	±*0.13*	±*0.47*	±*0.22*	±*0.17*	±*0.16*	
42	6.23abC	7.21bB	4.75aD	4.99aB	5.14aD	5.66c
	±*0.16*	±*0.143*	±*0.54*	±*0.26*	±*0.11*	
Means for experimental treatment	2.20A	2.54A	2.01A	2.08A	2.31A	
*Protease activity* (*PA*)						
7	123.59aAC	139.56aD	168.41aBC	245.55bC	159.16aBC	167.26b
	±*47.35*	±*12.82*	±*12.72*	±*4.59*	±*24.34*	
14	115.16aAC	127.58aCD	121.07aAB	264.04bC	241.71bD	173.91b
	±*24.41*	±*5.62*	±*3.26*	±*9.80*	±*6.94*	
21	75.22aAB	102.36aAB	109.16aA	175.51bB	161.90bC	124.83ab
	±*11.33*	±*8.04*	±*15.84*	±*19.96*	±*20.29*	
28	48.29aA	57.98abE	80.39bA	132.54cA	12.11cA	88.26a
	±*5.89*	±*2.63*	±*15.68*	±*16.16*	±*0.82*	
35	92.23aAB	84.24aA	89.93aA	119.30bA	130.0bAB	103.14a
	±*13.63*	±*4.35*	±*18.27*	±*8.81*	±*11.03*	
42	158.13abC	110.05aBC	180.61bC	164.56abB	119.37aA	146.55ab
	±*14.54*	±*13.49*	±*48.33*	±*17.90*	±*2.27*	
Means for experimental treatment	102.10A	103.63A	124.93A	**183.58B**	**155.71B**	

Explanations as for Table 3

The process of native keratin degradation by fungal strains was accompanied by increased extracellular keratinase activity. Its maximum activity was recorded towards the end of the experiment. Based on the analysis of curvilinear regression (R^2^ = 92.53%), small differences in keratinase activity were recorded between individual fungal strains. A statistical analysis of homogenous groups showed that the release of active keratinase did not differ significantly between the strains (Table 4).

### Accumulation of ammonium ions in the medium

A narrow C/N ratio encouraged the mineralization of nitrogen contained in feather keratin. The experiment showed that fungi periodically accumulated 321–592 µg of ammonium ions.cm^−3^ in the medium. The most intensive deamination was recorded in the first 4 weeks of fungal growth on feathers. The accumulation of ammonium ions decreased after 28 days of cultivation. Strains designated as 2, 3 and 4 mineralized N-keratin most actively, while strains 1 and 5 were the weakest amonificators (Table 5).

**Table 5:** Changes in the level of mineral products of keratin biodegradation: (A) ammonium ions (µg. cm^−3^) and (B) sulfate ions (mg. cm^−3^) in the post-culture liquids of *Chrysosporium* strains; values means of three replicates ± standard deviation

Times of analyses (days)	Strains of fungi					Means for time
	1	2	3	4	5	
*A. Ammonium ions*						
7	342. 45bA	468.52aD	498.97aB	440.37acA	395.84bcAB	429.23a
	±*22.86*	±*40.64*	±*21.79*	±*45.40*	±*7.33*	
14	496.66abB	528.27bcC	544.47cB	472.8aA	463.89aA	501.09b
	±*12.90*	±*8.74*	±*6.96*	±*14.41*	±*31.35*	
21	321.05aA	395.26bAB	423.79bA	341,29aB	322.01aA	501.09b
	±*18.68*	±*28.75*	±*32.25*	±*11.81*	±*12.86*	
28	472.76cB	563.16abC	551.79abB	592.27bC	425.91acAB	521.18b
	±*29.49*	±*19.83*	±*45.37*	±*54.25*	±*48.66*	
35	398.73aC	411.46aB	467.94bAC	440.37abA	437.87abA	431.27a
	±*13.22*	±*7.50*	±*14.48*	±*11.33*	±*36.01*	
42	336.47aA	358.25aA	422.25aA	409.53aAB	377.91aAB	380.88a
	±*14.06*	±*15.15*	±*39.06*	±*32.25*	±*105.41*	
Means for experimental treatment	**394,69B**	454,15A	484,87A	449,33A	**403,91B**	
*B. Sulfate ions*						
7	4,32aA	8.58aC	5.37aA	8.19aC	5.76aC	6.45c
	±*2.32*	±*0.52*	±*3.89*	±*0.79*	±*1.13*	
14	4,26abA	4.39abAB	4.72bA	3.86abA	3.27aAB	4.10a
	±*0.65*	±*0.40*	±*0.32*	±*0.46*	±*0.67*	
21	4,78aAB	4.78aA	4.85aA	3.80bA	3.14bA	4.27a
	±*0.65*	±*0.33*	±*0.19*	±*0.19*	±*0.56*	
28	5,44aAB	4.65abA	5.11aA	4.19abA	3.40bAB	4.56ab
	±*0.33*	±*0.52*	±*0.32*	±*0.67*	±*0.81*	
35	7.01bB	5.24aA	5.37abA	5.50abB	4.85aBC	5.59bc
	±*13.63*	±*4.35*	±*18.27*	±*8.81*	±*11.03*	
42	3.93aA	3.54abA	2.68bA	4.19aA	3.14abA	3.50a
	±*14.54*	±*13.49*	±*48.33*	±*17.90*	±*2.27*	
Means for experimental treatment	4.96AB	5.20AB	4.68AB	4.96AB	3.93A	

Explanations as for Table 3

### Accumulation of sulfate ions in the medium

Due to the high content of sulfur in feather keratin, sulfur mineralization products were also determined. Based on the results obtained in this study, strains were found to secrete up to 7.00 mg of S–SO_4_^2−^ between days 21 and 35 of cultivation but the maximum concentration of sulfate ions was recorded in the first week of fungal cultivation. Sulfate production decreased after this time and increased again from day 21 of fungal cultivation when proteolysis and ammonification were advanced. Sulfate release decreased slightly towards the end of the experiment (day 42). Statistically significant differences in the release of sulfate ions between the strains during culture were not detected (Table 5).

### Changes in the pH of the medium in cultures of fungi degrading feather waste

**Fig. 2:**
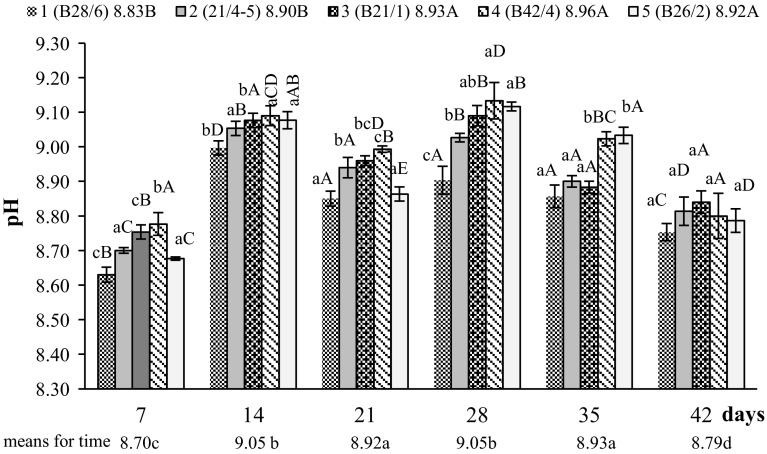
pH changes in the post-culture liquids of strains during biodegradation of feather waste; values means of three replicates ± standard deviation. Explanations as for Table 3

Investigations show that the medium became alkalinized up to pH = 9.13 (control pH = 7.30) during degradation of native feathers by all the strains. An increase in the pH was noticeable during rapid lysis, i.e. between days 7 and 14. Medium pH was stable in the next 2 weeks of cultivation. As the value of the coefficient of determination (R^2^ = 60.26%) showed, pH levels varied for individual fungal strains. This was also confirmed by statistical analysis of homogenous groups. Strains 3, 4 and 5 alkalinized the medium significantly more than the other two strains (Fig. 2).

### Determination of the main factor influencing the biodegradation of feather keratin by PCA (Principal Component Analysis) methods

**Fig. 3:**
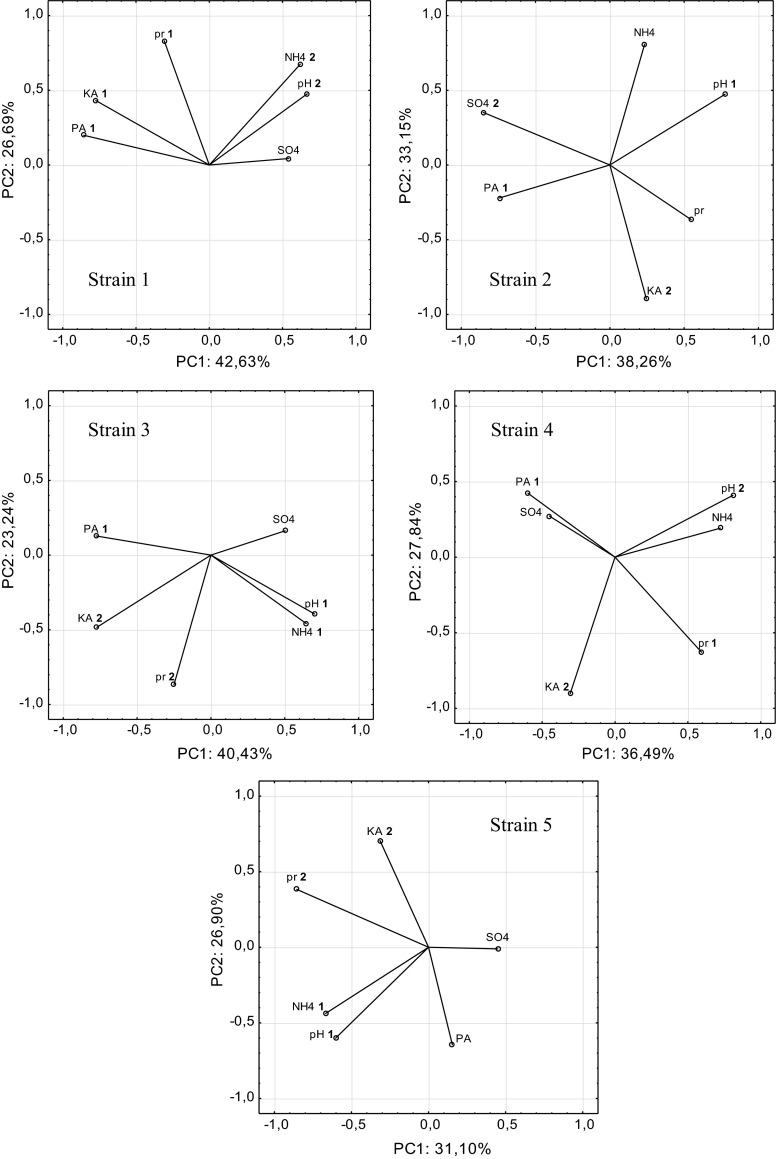
Results of principal component analysis (PCA) based on data of biochemical and chemical parameters in post-culture filtrate of five keratinolytic strains *PA* proteolytic activity; *KA* keratinolytic activity; *NH*4 ammonium ions; *SO*4 sulfate ions; *pH* pH, *pr* soluble proteins and peptides

Based on the criteria used in the Principal Component Analysis, main factors responsible for the biodegradation of feather keratin by *Aphanoascus fulvescens* strains and *Chrysosporium articulatum* strain were determined. Figure 3 shows the representation of the variables.

Biodegradation of feather keratin by the five strains (B28/6; B21/4–5; B25/1; B42/4; B26/2) was strong and amounted to 42.62; 38.26, 40.42, 36.48; 31.10% of total variance (TV%), respectively, and was conditioned by the release of proteins (pr) (80.30–86.49%), ammonium ions (NH4) (79.14–79.22%), keratinase activity (KA) (85.49%), protease activity (PA) (72.06–74.99%) and pH (80.25–90.59%).

During keratin waste biodegradation by strain 1 of *Aphanoascus fulvescens*, PC1 was more significantly involved in the biodegradation of feather keratin than PC2 and was strongly related to the release of peptides and proteins (pr) (0.803), keratinolytic activity (KA) (0.854) and proteolytic activity (PA) (0.749). Biodegradation of feather keratin by strain 2 of *A. fulvescens* was conditioned by PC1, which was most strongly related to proteolytic activity (PA) (0.729) and to the pH of the post-culture liquid (−0.905). Additionally, PC2 was positively related to keratinase activity (KA) (0.868) and negatively correlated with sulfate ions (SO4) (−0.788). During keratin biodegradation by strain 3 of C*hrysosporium articulatum*, PC1 was strongly related to ammonium ions (NH4) (0.791) and pH (0.802), and negatively correlated with protease activity (PA) (−0.72). According to the PCA, the two indices PC1 and PC2 explained 36.48 and 27.83% of total variance for strain 4 of *A. fulvescens*. Chiefly soluble proteins (pr) were positively correlated (0.8649) with PC1 while high protease activity (PA) was negatively correlated (−0.722) with it. PC2 was negatively related to pH (−0.860) and positively with KA (0.859). For strain 5 of *A. fulvescens,* PC1 showed that ammonium ions (NH4) (0.792) and pH (0.804) were highly correlated with biodegradation of feather keratin, and PC2 indicated keratinolytic activity (KA) (−0.773), the release of peptides and soluble proteins (−0.718).

Based on the results (Fig. 3), it may be accepted that biodegradation of feather keratin by all the strains under investigation was conditioned by the deamination of protein amino acids during which the cultures were alkalinized. However, the release of ammonium ion was negatively correlated with caseinian-protease and keratinase activities, which may indicate that another enzyme takes part in the deamination. Products of protein proteolysis and amonification may inhibit proteolytic activity (Korniłłowicz-Kowalska 1997a). The release of proteins and peptides was especially dependent on keratinolytic activity. Sulfate release in the majority of the strains (strains 2, 4 and 5) was related to protease activity. Keratinase activity (KA) was significantly positively correlated with protease activity only for strains 1, 2 and 3.

### Optimization of process variables using RSM

The Response Surface Methodology experimental design was applied for investigation of the relationship between process variables to optimize the loss of substrate mass by the *Aphanoascus fulvescens* B21/4-5 strain. The effect and nature of interactions of three process variables on feather mass loss were explored by a Box-Behnken design. Table 6 represents the Box-Behnken design matrix for the loss of substrate (feather) mass.

**Table 6:** The Box-Behnken design used for the three independent variables

Run	*X*_*1*_	*X*_*2*_	*X*_*3*_	Actual response (%)	Predicted response (%)		Run	*X*_*1*_	*X*_*2*_	*X*_*3*_	Actual response (%)	Predicted response (%)
1	−1	−1	0	48.40	45.87		24	0	−1	−1	37.60	33.09
2	1	−1	0	49.20	46.89		25	0	1	−1	3.80	3.27
3	−1	1	0	20.90	16.05		26	0	−1	1	42.90	49.96
4	1	1	0	9.60	17.07		27	0	1	1	21.60	20.14
5	−1	0	−1	23.00	28.63		28	0	0	0	62.00	64.77
6	1	0	−1	25.80	29.65		29	0	0	0	63.50	64.77
7	−1	0	1	37.00	45.51		30	0	0	0	69.60	64.77
8	1	0	1	50.35	46.52		31	−1	−1	0	44.40	45.87
9	0	−1	−1	34.80	33.09		32	1	−1	0	53.90	46.89
10	0	1	−1	2.40	3.27		33	−1	1	0	24.60	16.05
11	0	−1	1	42.20	49.96		34	1	1	0	18.90	17.07
12	0	1	1	22.10	20.14		35	−1	0	−1	42.40	28.63
13	0	0	0	67.00	64.77		36	1	0	−1	22.60	29.65
14	0	0	0	63.30	64.77		37	−1	0	1	47.00	45.51
15	0	0	0	65.10	67.77		38	1	0	1	51.75	46.52
16	−1	−1	0	32.40	45.87		39	0	−1	−1	38.20	33.09
17	1	−1	0	49.80	46.89		40	0	1	−1	4.80	3.27
18	−1	1	0	13.80	16.05		41	0	−1	1	53.70	49.96
19	1	1	0	11.80	17.07		42	0	1	1	15.35	20.14
20	−1	0	−1	23.40	28.63		43	0	0	0	67.50	64.77
21	1	0	−1	25.20	29.65		44	0	0	0	61.10	64.77
22	−1	0	1	50.95	45.51		45	0	0	0	63.90	64.77
23	1	0	1	51.55	46.52							

A multiple regression analysis represented the effect of the variables on the loss of substrate mass as the second order polynomial mathematical Eq. (3)

3 $$y_{\text{loss\,of\,substrate\,mass }}= R_{0} + R_{1} X_{1} + R_{2} X_{2} + R_{3} X_{3} + R_{12} X_{1} X_{2} + R_{13} X_{1} X_{3} + R_{23} X_{2} X_{3} + R_{11} X_{1}^{2} + R_{22} X_{2}^{2} + R_{33} X_{3}^{2} + \varepsilon$$

where *Y* is the response (loss of substrate mass), *X*_1_, *X*_2_, *X*_3_ are codded variables, *X*_1_^2^, *X*_2_^2^, *X*_3_^2^ are the square effects, *X*_12_, *X*_13_ are the interaction effects. *R*_*o*_- is a constant, *R*_1_ *, R*_2,_ *R*_3_ are linear coefficients, *R*_11_ *, R*_22_ *, R*_33_ are quadratic coefficients, *R*_12_ *, R*_13_ *, R*_23_ are cross product coefficients, and ɛ is a constant. Equation 4 represents the second order polynomial equation predicted for the loss of substrate mass

4 $$y_{{{\text{loss}}\;{\text{of}}\;{\text{substrate}}\;{\text{mass}}}} = - 1167.98 + 179.57X_{1} + 35.08X_{2} + 78.52X_{3} - 0.53X_{1} X_{2} + 3.14X_{1} X_{3} + 0.11X_{2} X_{3} - 11.13X_{1}^{2} - 0.54X_{2}^{2} - 37.67X_{3}^{2} + 40.60$$

Table 7 presents the relationships between the independent variables and dependent response in the form ANOVA. The analysis of variance of the regression model demonstrates that the model is highly significant. The coefficient of multiple regressions R^2^ was adjudged to be 0.9412 for the loss of substrate mass which indicates the fitness of the model. The adjusted R^2^ was calculated to be 0.9260 for the loss of substrate mass which indicates that the model is good for use in field conditions. The lack of fit is also significant because probability values are less than 0.0001.

**Table 7:** The analysis of variance of optimization experimental design for loss of substrate mass by keratynolytic strains

ANOVA for response surface quadratic model					
Source	Sum of squares	*df*	Mean square	*F* value	*p*
Model	15,786.23	9	1754.026	62.19	<0.0001*
X_1_	0.22	1	0.22	0.008	0.930
X_2_	5037.98	1	1364.19	48.37	<0.0001*
X_3_	1591.25	1	5037.98	178.64	<0.0001*
X_1_^2^	1364.19	1	2139.41	75.86	<0.0001*
X_2_^2^	2139.41	1	1591.25	56.42	<0.0001*
X_3_^2^	3738.37	1	3738.37	132.55	<0.0001*
X_1_X_2_	136.44	1	136.44	4.83	<0.0001*
X_1_X_3_	69.91	1	69.90	2.47	0.124
X_2_X_3_	3.68	1	3.68	0.13	0.720
Residual	987.06	35	28.20		
Lack of fit	189.19	3	63.06		
Pure error	797.87	32	24.93		
Cor total	16,773.29	44			

* Significant at 5% level; *R* ^2^ = 0.9411; $R_{\text{adj}}^{2}$ = 0.9260

### Effect of pH, temperature and the amount of feathers on the loss of substrate mass

The factors: temperature and amount of feathers, are important for the loss off substrate mass under experimental condition (Table 7). The predicted percentage of the loss of substrate mass was 64.77% which was in agreement with 61.10–69.60% obtained experimentally with 28 °C, pH 7.5 for the concentration of 1.0 g feathers(Table 6). The maximum loss of substrate mass (71.01%) is obtained for critical values: pH = 7.58, temperature 28.7^o^ C and 1.4 g of feathers on 100 ml of the medium (Table 8).

**Table 8:** Critical values

	Observed minimum	Critical value	Observed maximum
pH	6.5	7.58	8.5
Temperature (^o^C)	25	28.70	37
Feathers (g)	0.5	1.40	2
Predicted value (%)	71.08		

**Fig. 4:**
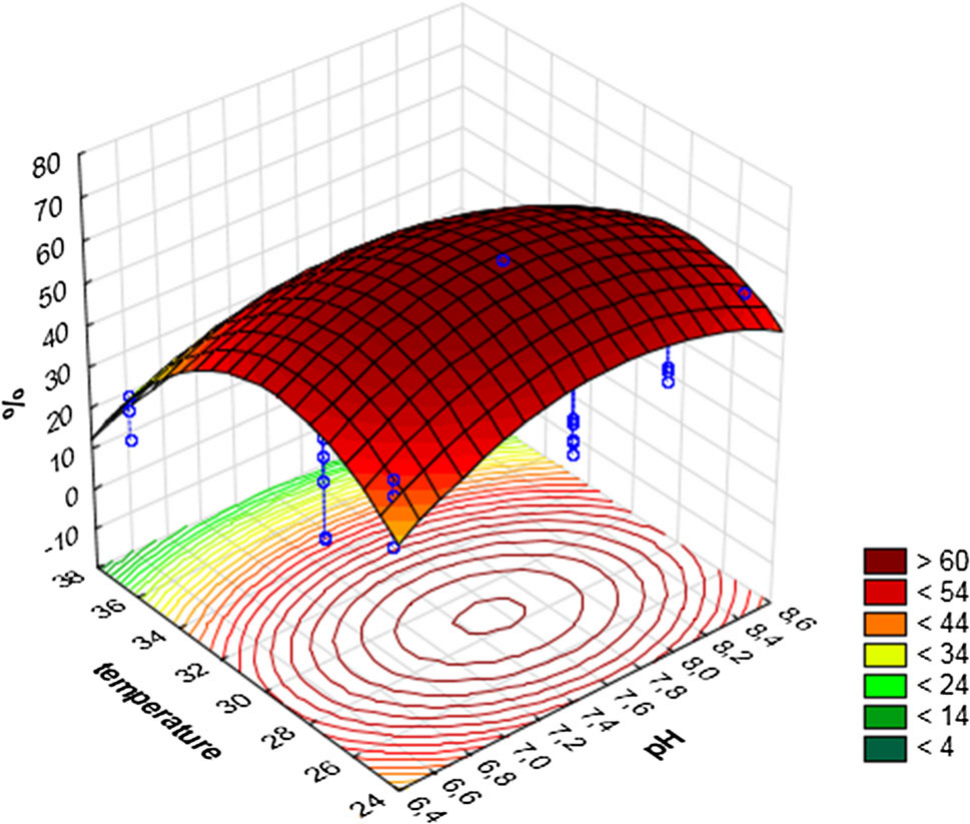
Loss of substrate mass (%) on 3-D graphics for response surface optimization versus pH and temperature

**Fig. 5:**
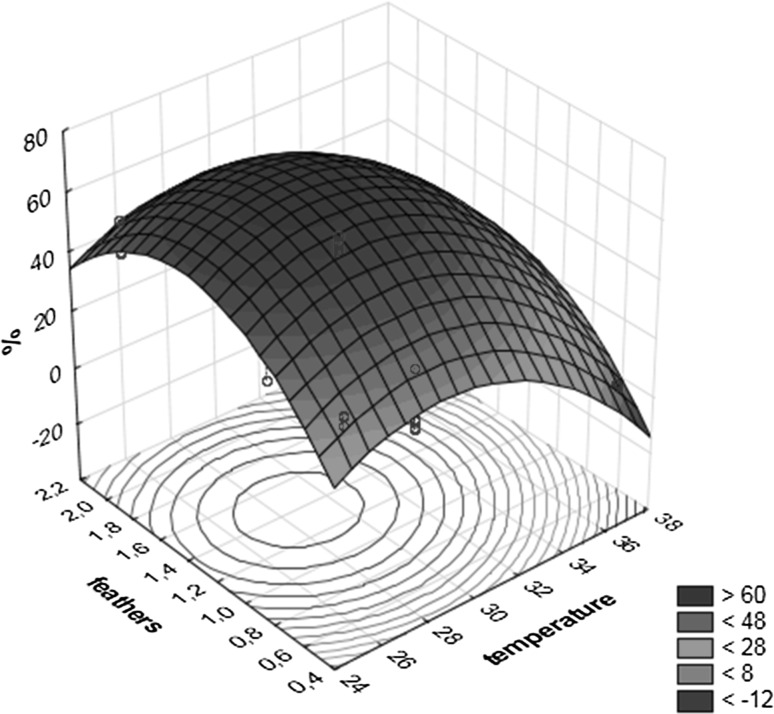
Loss of substrate mass (%) on 3-D graphics for response surface optimization versus feathers and temperature

The relationships between variables and the response effects are illustrated in two-dimensional plots. Figure 4 shows effects of pH and temperature on the loss of substrate mass (%) due to a significant interaction between them. The second dimensional plot (Fig. 5) presents the effect of the loss of substrate mass with respect to temperature and the amount of feathers.

## Discussion

Liu and Paterson (2011) recognize the analysis of selected nucleotide sequences of rRNA genes as an unambiguous and rapid method for identification of fungi of the genus *Chrysosporium*. To date, species identification of keratinolytic fungi belonging to the genus still poses several challenges both for phenotypic traits as shown by Vidal et al. (2000) and for molecular (genetic) methods. The nomenclature of species (anamorph and teleomorph) is not homogenous in databases and collections, and the sequences of DNA fragments obtained in this study were placed in different species when compared to a range of databases. Both this and the author’s studies show that it is necessary to examine morphological and genetic characters for species identification of fungi belonging to the genus *Chrysosporium*.

Five strains were examined in this study. Based on observations of macro- and microphorphological characters as well as an analysis of ITS 1 and ITS 4 fragments, four strains (no 1-B28-6; 2-B21/4-5; 4-B42/4; 5-B26/2) were identified as *Aphanoascus fulvescens* (teleomorph of *Chrysosporium keratinophilum* according to the ATCC strain collection) and one strain (no 3-B25/1) was identified as *Chrysosporium articulatum.*

Keratinophilic fungi of the genus *Chrysosporium* participate in the recycling of carbon, nitrogen, sulfur and energy of native keratin in the soil and in other environments containing keratin residues (Kushwaha 2000). For those reasons the fungi constitute an important group among the destruents of organic matter in the soil and, as reported by Singh (2002), in the bioremediation of environments polluted with keratin waste. According to Vasileva-Tonkova et al. (2009) and Syed et al. (2009), microbiological degradation of keratin waste is also an ecologically and economically safe method of utilization of such waste. Brandelli et al. (2010, 2015) and Lasekan et al. (2013) believe that products of microbiological keratinolysis and their enzymes can have industrial applications.

Like fungi belonging to dermatophytes, keratinolytic saprotrophic fungi are able to fully degrade native keratin substrate (Filipello Marchisio et al. 1994). The following factors participate in the keratinolysis of dermatophytes: the mechanical factor, i.e. perforating organs responsible for the destruction of native keratin, and the enzymatic factor responsible for the lysis of this substrate (English 1968; Kunert 1989; Filipello Marchisio 2000). English (1969) reports that non-dermatophytic keratinolytic fungi of the genus *Chrysosporium* exhibit keratinolytic abilities against hard keratin; however, unlike dermatophytes, they do not produce perforating organs but only swollen hyphae known as boring hyphae. Different data regarding morphological traits of in vitro keratinolysis by strains of the genus *Chrysosporium* was obtained by Mitola et al. (2002). According to their results, keratinolysis by species of the genus *Chrysosporium* is related to the surface erosion of hair, defibrement, radial penetration and the activity of perforating organs. As reported in a study by Korniłłowicz-Kowalska and Bohacz (2011), fungi producing perforating organs are strong destruents of native keratin quickly degrading this substrate. The utilization of native keratin as the substrate is important for the assessment of keratinolytic abilities of microorganisms. Korniłłowicz-Kowalska (1997a) believes that this is measurable by the loss of substrate mass (native keratin). Kumar et al. (2015) show that fungi of the genus *Chrysosporium* cause the loss of substrate (feather) mass ranging from 68 to 82%. The present author’s investigations show that strains of *Aphanoascus fulvescens* and *Chrysosporium articulatum* biodegraded native chicken feathers in 47.37–65.90%. These values as twice as high as those obtained for the reference strain, CBS 104.62. Based on this, the strains under consideration can be classified as having strong keratinolytic abilities. As defined by Kunert (2000), microorganisms which degrade more than 40% of keratin after 8 weeks in liquid culture are strongly keratinolytic.

The activity of keratinase is recognized as a reliable indicator of keratinolytic abilities of microfungi in the majority of studies (Anbu et al. 2005, 2007; Liang et al. 2011). Liang et al. (2011) report that based on the regression model for keratinase activity of *Myceliophtora thermophila* the optimum of cultivation conditions is pH 7.9, 20.10 g l^−1^ chicken feathers and 0.98 g l^−1^ urea. Anbu et al. (2005) report that the maximum of keratinase activity calculated using a Box-Behnken design ranges within pH 6.5–7.9 and temperature 25.0–47.5 °C. The optimization of the medium composition and culture conditions for the loss of substrate mass by fungi of the genus *Aphanoascus* has not been reported was not reported. In this study by applying a Box-Behnken desing a considerably smaller combination of factors and levels was used for effectively studying the optimum condition for loss of feathers mass. Box-Behnken designs obtained in this study show that for *Aphanoascus* B21/4-5 the maximum loss of substrate mass (71.08%) is achieved for pH 7.58 and temperature 28.7 °C (Table 8). Optimum conditions where 64.77% feather mass loss is achieved are recorded for 28 °C, pH 7.5 and 1 g feathers on 100 ml of the medium (Table 6).

As well as the loss of substrate mass, criteria of microbiological keratinolysis include the release of peptide substances, amino groups, amino acids, ammonium, sulfhydryl groups, the secretion of sulfates, keratinase activity and alkalinization (Korniłłowicz 1994; Korniłłowicz-Kowalska 1997a; Kunert 2000). Singh (2002) reports that the degree of keratin degradation correlated with the changes in enzymatic activity is the direct evidence of keratinolytic abilities of fungi. Investigations conducted in the present paper show that the biodegradation of chicken feathers by five different strains occurs with active keratinases and proteases. As shown in Fig. 3, the release of proteins and peptides was correlated with keratinase activity but not with extracellular caseinian protease activity of these microorganisms. The loss of chicken feather mass was the highest after cultivation of the *A.fulvescens* B 21/4-5 strain. In this period (day 42) keratinolytic activity was the highest not only for this strain but also for the remaining strains (Fig. 1). As observed by Korniłłowicz-Kowalska (1999), raw treated preparations of fungal keratinases do not fully solubilize hard keratin. As well as Grzywnowicz et al. (1989) and Łobarzewski et al. (1990), Korniłłowicz-Kowalska (1997a) indicates that keratinolytic activity is conditioned by the presence of different proteases, including keratinolytic proteases. Strains examined in this study produced active proteo- and keratinolytic enzymes as evidenced by the changes of these enzymes during cultivation and a generally positive correlation between the activities of proteases and keratinases (strains 1, 2, 3). According to Korniłłowicz-Kowalska (1997a), a "cascade” of enzymes with affinity to various keratin proteins acts in cultures of geophilic dermatophytes and *Chrysosporium*. These observations could be supported by the present results which show that keratinase activity is preceded by caseinian protease activity. The maximum of keratinase activity in the cultures was on day 42 but the maximum activity of caseinian protease was after 7 or 14 days for the majority of strains. The maximum activity of keratinase in week 5 of *Scopulariopsis brevicaulis* cultivation at 30 °C in pH 7.5 was also observed by Anbu et al. (2007). As shown in Fig. 3, protease activity is related to sulfate ions whose concentration was the highest in the first week of cultivation and on day 35. The activity of extracellular keratinases secreted in the cultures of the strains did not differ significantly between the strains and had similar, quite low values. Studies by several authors (Sousa et al. 2015; Anbu et al. 2007) show that fungi of the genera *Aspergillus*, *Acremonium*, *Peacilomycs, Trichoderma* and *Scopulariopsis* also secrete active keratinase against feather keratin as the substrate and their activity is similar to that recorded in the present study. However, the low activity of keratinases in cultures of keratinolytic fungi (*Chrysosporium* and *Trichophyton*) does not always preclude their keratinolytic abilities as mechanical destruction is the second important constituent of keratinolysis, as strongly emphasized by Mitola et al. (2002). Only these two factors, i.e. strong mechanical destruction combined with lysis, produce the effect of keratinolysis (Kunert 2000; Mitola et al. 2002). The reserve effect was noted in studies by Singh (2002) where a high activity of fungal keratinases was correlated with weak solubilization of keratin substrate. The authors attribute this to the removal or repression of some accessory proteins required by keratinase to act effectively on the keratin molecule while splitting its disulfide bonds.

Due to the high content of nitrogen (15–18%) and sulfur (3–5%), inorganic nitrogen and sulfur products (ammonium ions and sulfate ions) are released during biodegradation of native feather keratin. After 42 days of cultivation *A. fulvescens* strains and *Chrysosporium articulatum* strain caused solubilisation and mineralization of feather proteins as indicated by the release of peptides and the accumulation of N-NH_4_^+^ in the medium correlated with increased pH reaching 8.6–9.1 (alkalinization). As reported by Sanyal et al. (1985) and Elíades et al. (2010), an alkaline reaction is favourable for the production of keratinolytic protease by fungi. Korniłłowicz-Kowalska (1997a) demonstrated that saprotrophic keratinolytic fungi, represented mainly by a variety of species of geophilic dermatophytes and *Chrysosporium*, converted up to 75% of nitrogen into the ammonium form during full solubilization of native feather keratin. Remaining nitrogen was released as low- and high-molecule peptides and amino acids (Korniłłowicz-Kowalska 1997a, b). The present author’s studies show that no more than 20% of total nitrogen of feather keratin in cultures of all strains is released as peptides and amino acids and between 26% and 46% as ammonium ions. The release of ammonium ions in the cultures of *A. fulvescens* and *Chrysosporium articultaum* strains was negatively correlated with the activity of protease. It may therefore be suspected that proteolytic enzymes other than caseinian protease take part in the release of ammonium ions from simple proteins and amino acids, that this enzyme is inhibited by the high concentration of ammonium ions or, as discussed by Korniłłowicz-Kowalska (1997a), a factor other than enzymatic plays an important role. Products of protein proteolysis and amonification may inhibit proteolytic activity (Korniłłowicz-Kowalska 1997a). In the present study, a decrease in the concentration of ammonium ions at the end of the experiment (i.e. after 28 days of cultivation) caused a significant increase in the activity of keratinase and a smaller increase in the activity of caseinian protease. Studies by Kunert (2000) indicate that inorganic sulfite secreted by the fungus is an important constituent of keratin biodegradation by keratinolytic fungi. It participates in the sulfitolysis, that is the cleaving of disulfide bonds of native keratin, leading to the relaxation of the protein’s structure, which consequently enables keratinolytic proteases to attack and cystine’s surplus sulfur to be released as sulfates and sulfocysteine. The onset of sulfitolysis is conditioned by the alkalinization of the reaction’s environment (Kunert 2000). Studies by Williams et al. (1990) demonstrate, however, that sulfur amino acids are the proper product of catabolism of keratin sulfur by keratinolytic bacteria. The highest sulfate release and protease activity occurring at the same time, noted in the present study, suggests that these processes may occur in parallel. This suggestion is supported by the results obtained by Ruffin et al. (1976) conducted in cultures of keratinolytic fungi. They show that sulfitolysis occurs in parallel with proteolysis of native keratin. The strains of *A. fulvescens* and *Ch. articulatum* secreted from 2.68 to 8.58 mg of S-SO_4_^2−^, which corresponded to 7–24% of S-feather. As only small amounts of sulfates were detected in cultures of prokaryotic microorganisms, i.e. *Streptomyces fradiae,* degrading native wool in mineral medium (Kunert 1989), different keratinolysis mechanisms occur in Procaryota and Eucaryota and different applications of these microorganisms can be proposed.

## Conclusions

1. Based on the results, all the strains of keratinophilic fungi under consideration exhibit keratinolytic activity against native feather keratin. This is supported by numerical values of coefficients of keratinolytic activity, i.e. the loss of keratin substrate mass, the release of soluble proteins and peptides, N–NH_4_^+^, S–SO_4_^2−^, changes in the pH and keratinase activity.
2. The Principal Component Analysis showed that the biodegradation of native feather keratin by strains of *A. fulvescens* and *Ch. articulatum* took place with active proteolytic enzymes. The activity of protease with keratinolytic properties (keratinases) is significantly correlated with the release of proteins and peptides. The release of a large number of ammonium ions causes a significant increase in the pH of the medium. The accumulation of a large amount of ammonium ions slows down the activity of caseinian protease and keratinase activity (a negative correlation between the activities of caseinian protease and keratinase activity and ammonium ions) while a decrease in these ions encourages the induction of active keratinases and caseinian proteases and a significant increase in released peptides and sulfates, products of fungal keratinolysis.
3. The greatest keratinolytic activity was detected for *Aphanoascus fulvescens* B21/4-5. High levels of keratinase activity and the highest percentage values of the loss of keratin substrate mass the release of high amounts of proteins and the accumulation of S-SO_4_^2−^ and N–NH_4_^+^ ions were recorded during culture. This addresses the current interest in the potential application of this microorganisms as natural agents enriching soils poor in mineral nitrogen and sulfur.
4. This study shows that statistical optimization tools and Response Surface Methodology are helpful in finding the optimum level parameters (significant variables) for the loss of substrate (feathers) mass (%). The optimization of cultivation conditions of strain B21/4-5 using a Box-Behnken design against the loss of substrate mass (a 300 ml Erlenmeyer flask and 100 ml of the medium) showed that the most favourable conditions for the degradation of feathers are recorded at pH 7.58, 28.7 °C and 1.4 g of sterile chicken feathers for fungi belonging to *Aphanoascus fulvescens* (teleomorph of *Chrysosporium keratinophilum*).
5. When the level of mineral products of the biodegradation of native keratin is known, keratin waste management using these fungi can be adequately established and selected. Due to the accumulation of ammonium and sulfate ions in the cultures of *A. fulvescens* and *Ch. articulatum* strains, high-efficiency strains of this species can potentially be used as natural biofertilization factors.
